# The Proceedings of the American Society of Dental Surgeons

**Published:** 1845-09

**Authors:** 


*The Proceedings of the American Society of Dental Surgeons.*—We regret that we are unable to publish, in this number, the very excellent opening address, delivered by Dr. J. H. Foster, at the last meeting of the Society. We are glad, however, to be able to announce that we are promised a report of it for the next. We are also sorry that we are compelled to omit, for want of room, two of the dissertations delivered on that occasion, but they shall appear in the December number. For the transactions of the Society at its last meeting, the reader is referred to the minutes of the Recording Secretary, in another part of this number.

